# Doctor At Work, Late Evening

**Published:** 2007

Everyone has gone home
but you and the janitor. The nurses
have driven away, fluttering across the parking lot
like white confetti.

Sparrows sing themselves to sleep and even the hospital
longs to dream. You read X-rays
in the view box glow, your patients′ names
marked in black, their lungs
surprised in full inhale, hearts stopped
mid-pump, strangers
who keep you company as you scan for ribs
cracked like barrel staves, for emphysema's
burst balloon, for pinpoint tumours
light years away, only beginning to burn.

The janitor knocks.

You close the light, wishing each patient safe,
every diagnosis exact. Outside,
stars ignite, like cancers inflaming the sky.
Cortney Davis

